# Dentigerous Cystic Changes in the Follicles Associated with Radiographically Normal Impacted Mandibular Third Molars

**DOI:** 10.1155/2018/2645878

**Published:** 2018-03-20

**Authors:** Ashok Dongol, Alok Sagtani, Mehul Rajesh Jaisani, Arpita Singh, Ashish Shrestha, Anju Pradhan, Pradeep Acharya, Anjani Kumar Yadav, Ram Prasad Yadav, Arun Kumar Mahat, Iccha Kumar Maharjan, Leeza Pradhan

**Affiliations:** ^1^Department of Oral and Maxillofacial Surgery, BP Koirala Institute of Health Sciences, Dharan, Nepal; ^2^Patan Academy of Health Sciences, Kathmandu, Nepal; ^3^B & C Hospital, Birtamod, Nepal; ^4^Department of Oral Pathology, BP Koirala Institute of Health Sciences, Dharan, Nepal; ^5^Department of Pathology, BP Koirala Institute of Health Sciences, Dharan, Nepal; ^6^Department of Oral Medicine and Radiology, BP Koirala Institute of Health Sciences, Dharan, Nepal; ^7^Kathmandu Model Hospital, Kathmandu, Nepal

## Abstract

**Objective:**

To assess the incidence of dentigerous cystic changes in the follicles of radiographically normal impacted mandibular third molars.

**Methods:**

One hundred and thirteen follicles obtained after surgical removal of impacted mandibular third molars with radiolucency of less than 2.5 mm in the radiograph were sent for histopathologic evaluation to evaluate pathologic changes.

**Results:**

The incidence of dentigerous cystic changes observed was 15.9%, that is, 18 out of 113 patients (51 males and 62 females), with the maximum incidence of cystic changes seen in the follicular space size of 0.5 mm. The mean age of the patients included was 27.8 ± 8.1. The most common indication for extraction among the patients in this study was recurrent pericoronitis (95%). There were no statistically significant differences in occurrence of cystic changes based on age, gender, angulation, relation to ramus, depth, side of impaction, and follicle size (*P* > 0.05).

**Conclusion:**

Dental follicles obtained from surgically removed impacted mandibular third molars should be submitted for histopathologic examination irrespective of the radiographic size of the follicle.

## 1. Introduction

Impacted mandibular third molars are common among patients seen in the oral and maxillofacial surgery [1]. Infection, nonrestorable caries, cysts, tumors, and destruction of the adjacent teeth and bone are considered as indications for the removal of impacted third molars [2]. However, prophylactic extraction of the asymptomatic impacted third molar is routinely practiced in Europe and the United States. The justification for prophylactic extraction includes the need to minimize the risk of pathologic changes like cysts and tumors, increased difficulty of surgery with age, and reduction of the risk of mandibular angle fracture, and that the third molars have no definite role in the mouth [3]. The prophylactic extraction is still controversial.

The dental follicle is an ectomesenchymal tissue that surrounds the developing tooth germ. In a radiograph, it is seen as a normal homogeneous radiolucent space around the crown of a developing tooth and is known as the follicular space. The dental follicle associated with the impacted third molar has the potential to undergo cystic degeneration and form dentigerous cyst, odontogenic keratocyst, and ameloblastoma [4]. Follicular space of size less than 2.5 mm in width is considered as radiographically normal or not being associated with any pathology [5]. However, this may not be a reliable method to know the pathology associated with impacted teeth. So, it would be prudent to do a histological examination of the follicle associated with the impacted teeth as the absence of radiographic disease may not necessarily indicate the absence of the disease. The incidence of the pathological changes associated with impacted teeth seems to be higher than that reported from the radiographic studies [4, 6–9]. The removal of impacted teeth followed by the histopathologic examination of the follicle associated can thus decrease the chances of development of cysts and tumors and alleviate extensive treatment [3].

Therefore, the present study was conducted to assess the incidence of pathologic changes in the follicle of radiographically normal impacted mandibular third molars and to evaluate the association of cystic changes in the follicle with the patient's age, sex, angular position, and contact of the impacted tooth with adjacent tooth.

## 2. Materials and Methods

**Figure 1:**
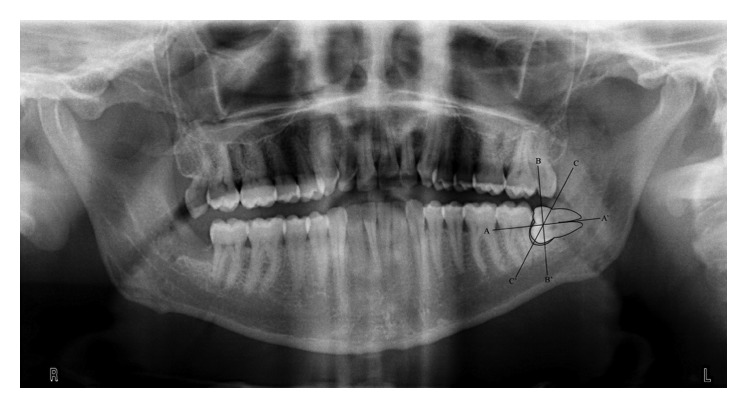
Follicle size measurement.

One hundred and thirteen patients aged 16–60 years with impacted mandibular third molars with a follicular space of less than 2.5 mm in the panoramic radiograph were included in the study. For radiographic measurement of the follicular size, contours of the impacted mandibular third molar and pericoronal radiolucency were traced on the tracing paper on the X-ray viewer. A straight line (AA′) passing through the long axis of the impacted tooth was drawn, and the other line (BB′) was drawn perpendicular to AA′ and passing through the centre of the crown. The widest point of the follicular space was measured as per the method reported by Damante and Fleury [10]. Another straight line (CC′) was drawn from the intersection of the two lines (AA′ and BB′) to the widest area of the follicular space. Along this line (CC′), the follicle size was determined by measuring the distance between the contour of the tooth and the widest area of the follicle using a caliper ruler (Figure 1).

**Figure 2:**
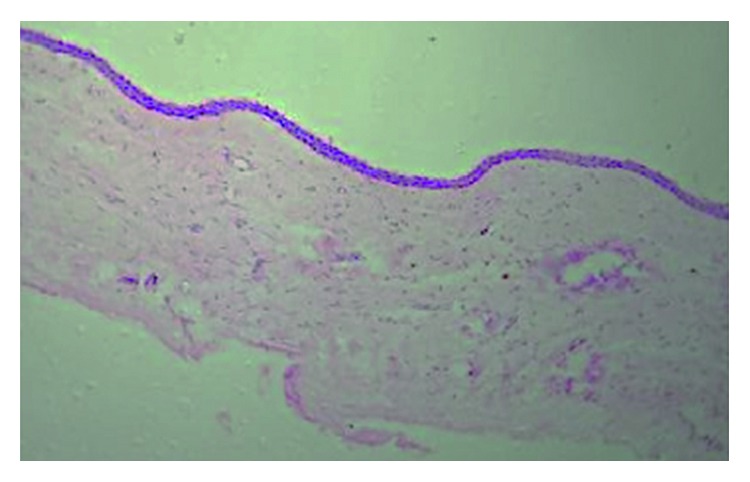
Lining of the nonkeratinized stratified squamous epithelium with odontogenic islands suggestive of dentigerous cyst (H&E stains; 10x).

**Figure 3:**
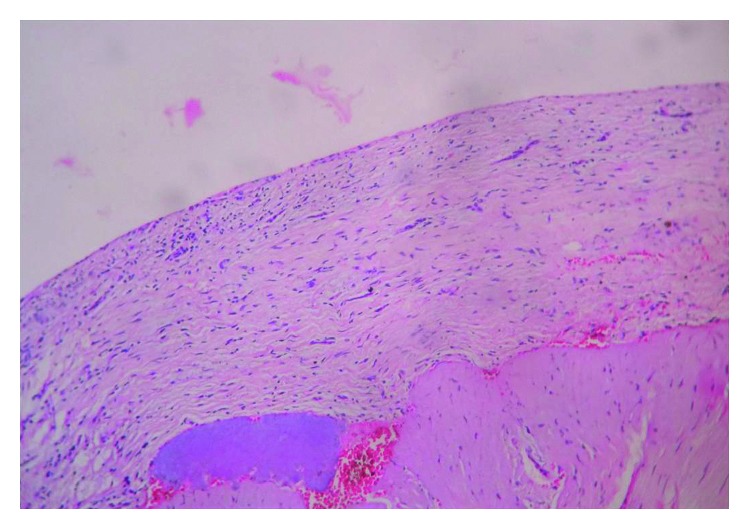
Dental follicle devoid of epithelial lining (H&E stains; 10x).

After complete extraction of the tooth, follicular tissue around the crown of the tooth was collected in 10% neutral buffered formalin and sent for routine histopathological examination and stained with hematoxylin and eosin. One hundred and thirteen follicles thus obtained were sent for histopathologic evaluation to evaluate pathologic changes. A pericoronal radiolucency with a histopathologically continuous lining of the nonkeratinized stratified squamous epithelium and a cystic space between enamel and overlying tissue was considered as a dentigerous cyst (Figure 2), whereas tissue devoid of epithelial lining with no epithelial cell rests was considered as the normal follicle (Figure 3).

The association between the follicle and pathologic changes, age, gender, angular position, and contact of the impacted tooth with adjacent tooth was statistically evaluated. The measurement of association of categorical variables was done through the chi-square test and calculation of odds ratio with 95% confidence interval.

The study was conducted in accordance with the Declaration of Helsinki. Ethical approval was obtained from the Institutional Ethical Review Board, BP Koirala Institute of Health Sciences. Informed written consent was taken from the patients.

## 3. Results

**Figure 4:**
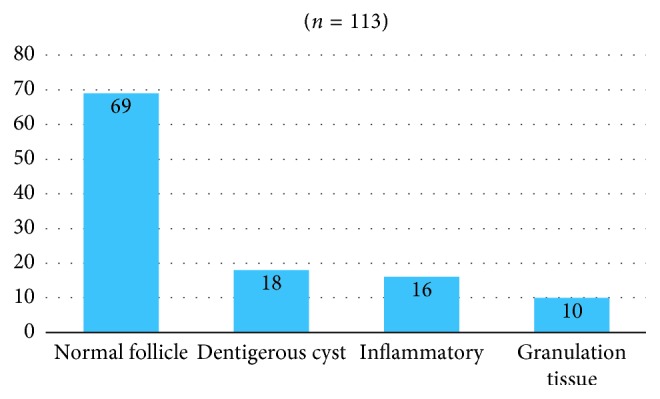
Frequency distribution of histopathological diagnosis.

**Figure 5:**
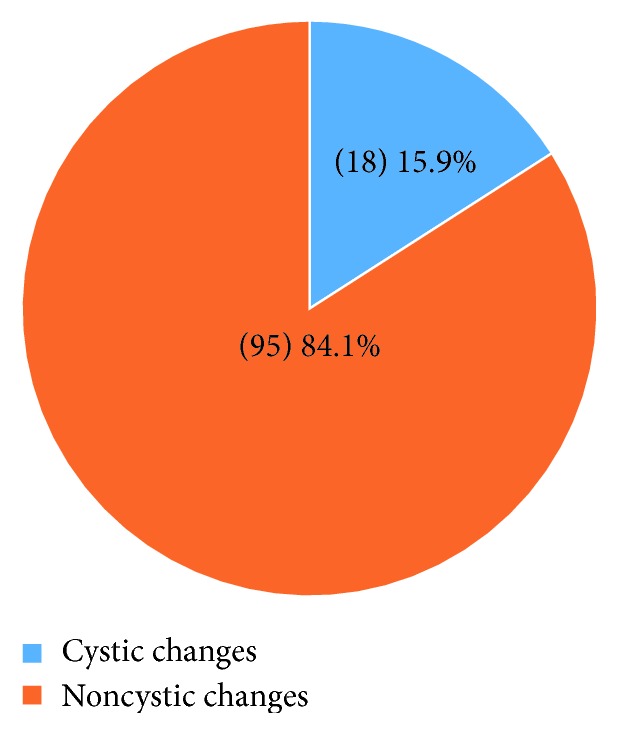
Frequency distribution of cystic changes.

Of the 113 follicle tissues studied, 51 (45.1%) were from male patients and 62 (54.9%) from female patients. The age of the patients ranged from 16 to 53 years with the maximum number of patients in the range between 26 and 30 (*n*=32) followed by 21- to 25-year (*n*=30) age group. The mean age of the patients was 27.8 years with the standard deviation of ±8.1. The most common cause of extraction of the impacted mandibular third molar was recurrent pericoronitis which was seen in 95 patients (84.1%), followed by carious third molar and carious second molar in 7 patients each (6.2%) and prophylactic in 4 patients (3.5%). Normal follicle (69 patients, 61.1%) was the most common histopathologic finding with only 18 patients (15.9%) showing dentigerous cyst, as shown in Figure 4. Considering the normal follicle, inflammatory tissue, and granulation tissue as noncystic changes, 18 (15.9%) had cystic changes and 95 (84.1%) showed no cystic changes which is suggestive of less incidence of cystic changes in the follicle around the impacted third molar (Figure 5).

There was no statistically significant difference (*P*=0.95) in the occurrence of cystic changes based on gender. Ten out of 62 female patients and 8 out of 51 male patients had cystic changes. There was no statistically significant difference in the occurrence of cystic changes among the patients in relation to age (Table 1). Cystic changes were seen in tissues from patients as young as 16 and as old as 42, and the mean age of the patients with cystic changes was found to be 27 years.

No statistical significance was observed in the occurrence of cystic changes based on the angulation pattern of the impacted tooth (*P*=0.816), the depth of impaction (*P*=0.826), and relationship of the impacted tooth with the ramus (*P*=0.442), on side distribution of the impacted mandibular third molar as shown in Table 2.

Upon evaluation of association of dentigerous cystic changes with the follicle size, cystic changes were observed more in the follicle size of 0.5 mm as shown in Table 3. No statistically significant difference was found in the occurrence of cystic changes based on the follicle size (Table 4).

## 4. Discussion

Impacted mandibular third molars are routinely indicated for extraction when signs and symptoms of pathosis are present. But there is still no general agreement on whether to remove asymptomatic third molars or not. As the incidence of systemic diseases, pathological conditions associated with the impacted tooth, and postsurgical morbidity increases with age, so do the difficulty and complications of impacted third molar surgery [3]. These causes support the prophylactic removal. Proponents of prophylactic removal have also suggested high incidence of cystic changes associated with impacted third molars with the follicular space even less than 2.5 mm, which is considered radiographically normal [5]. Baykul et al. [6] found 50%, Saravana and Subhashraj [7] found 46%, Glosser and Campbell [8] found 37%, Rakprasitkul [4] found 35%, Adelsperger et al. [9] found 34%, and Yıldırım et al. [11] found 23% dentigerous cystic changes in impacted third molars. Besides dentigerous cysts as the detected pathologic entity, others have also reported odontogenic keratocyst, calcifying odontogenic cysts, ameloblastoma, myxoma, and odontogenic fibromas [12–15].

Therefore, the present study was carried out to evaluate the pathologic changes associated with the normal radiographic follicular size in mandibular impacted third molars. Glosser and Campbell [8] and Curran et al. [12] suggested that any follicle with the stratified squamous epithelium should be regarded as a dentigerous cyst. Cystic changes observed in our study were only of dentigerous cyst with the nonkeratinized stratified squamous epithelium in 15.9% of the follicles. The value observed in the present study is lower than those reported by the aforementioned authors. But still, this observation should alert the surgeons to submit the follicles obtained after extraction for histopathologic evaluation. Less percentage of cystic changes in this study is probably because the pathologic condition may have involuted and not progressed to detectable lesion as the mean age of the patients is higher than that compared to other studies. So with age-related changes, the tissue could have undergone conversion to a quiescent state, persisting only as a histologic aberration of little clinical significance [16]. Thus, the patients with cystic changes should be kept for long-term follow-up to assess the progression of the disease. This will eventually help in the early detection of pathology if any related to cystic changes followed by its management and thus may prevent morbidities associated with the cyst. Also, the added cost of diagnosis based on the pathologic evaluation is worth than the risk of future deterioration from cystic changes.

The mean age of the patients with cystic changes was found to be 27 years in our study with the occurrence of cystic changes from 16 years to 42 years of age. Studies have suggested that groups older than the second decade show higher incidences of pathologic changes [8, 9]. Histopathologic diagnosis of cystic changes showed a female-to-male ratio of 1.25 : 1 in our study considering the fact that the incidence of impaction is more in females [17]. Other studies have observed male predominance with cystic changes, but the reason for this gender difference is still unknown [4, 9, 18]. The most common cause for extraction among the patients in this study was recurrent pericoronitis (84.1%). In the context of our country, patients rarely seek dental treatment when the tooth is asymptomatic. The alarming symptoms of acute and/or recurrent pericoronitis might be the convincing reason for the patients to seek for immediate dental treatment and be motivated for extraction.

A significant difference could not be found regarding the relationship between dentigerous cystic changes and depth of impaction as well as the relation of the impacted third molar with ramus in our study. Although no statistically significant association between cystic changes and angulation of the third molar was observed, higher probability of cystic changes was found in vertically positioned impacted third molars (21.4%) as observed in the study by Baykul et al. [6]. However, Knutsson et al. [17] and Eliasson et al. [5] reported higher incidence of pathologic changes in horizontally positioned impacted third molars, while Adaki et al. [16] found higher probability of cystic changes in the distoangular impaction. Such differences observed may be due to different study designs with different inclusion criteria. But the variation also suggests that all types of the angulation pattern of impaction can potentially be associated with cystic changes.

## 5. Conclusion

Cystic changes may be found in small follicular spaces, whereas there may be histologically normal tissues in big radiolucent lesions [19]. Therefore, according to the results of this study, dental follicles of surgically removed impacted third molars should be submitted for histopathologic examination irrespective of the follicle size because dentigerous cystic changes occurring in the follicle tissue cannot be disregarded. There should also be a standard follow-up protocol for periodic evaluation of the patients with evidence of cystic changes to detect any further clinical or pathologic changes so as to prevent morbidity associated with it.

## Figures and Tables

**Table 1:** Relationship between dentigerous cystic changes and age.

Sociodemographic characteristics	Cystic changes	Noncystic changes	*t*-test	Lower 95% CI	Upper 95% CI	*P* value
Age (years)	27 ± 7.4	27.9 ± 8.3	0.44	−5.08	3.23	0.66

**Table 2:** Relationship between dentigerous cystic changes and angulation, depth, and relation of the impacted tooth to ramus.

Parameters related to impaction		Number (*n*=113)	Dentigerous cyst		*P* value
			Present	Absent	
Angulation of the impacted third molar	Mesioangular	52	7 (13.5%)	45 (86.5%)	0.816
	Horizontal	25	4 (16%)	21 (84%)	
	Vertical	28	6 (21.4%)	22 (78.6%)	
	Distoangular	8	1 (12.5%)	7 (87.5%)	

Depth of impaction	Position A	37	5 (13.5%)	32 (86.5%)	0.826
	Position B	68	12 (17.6%)	56 (82.4%)	
	Position C	8	1 (12.5%)	7 (87.5%)	

Relationship of the impacted tooth with ramus	Class I	41	7 (17.1%)	34 (82.9%)	0.442
	Class II	64	11 (17.2%)	53 (82.8%)	
	Class III	8	0 (0%)	8 (100%)	

Side distribution	Left	51	9 (17.6%)	42 (82.4%)	0.65
	Right	62	9 (14.5%)	53 (85.5%)	

**Table 3:** Incidence of dentigerous cyst according to the follicular size.

Follicular size (mm)	Number of patients (*n*=113)	Dentigerous cyst
0.5	12	3 (25%)
1	45	7 (15.5%)
1.5	18	2 (11.1%)
2	28	4 (14.3%)
2.5	10	2 (20%)

**Table 4:** Relationship between dentigerous cystic changes and follicle size.

	Cystic changes	Noncystic changes	*t*-test	Lower 95% CI	Upper 95% CI	*P* value
Follicle size (mm)	1.36 ± 0.66	1.42 ± 0.58	3.58	−0.36	0.25	0.72
